# Quantitative investigation on working memory patterns through EEG based on visual attention task for children with learning disability

**DOI:** 10.3389/fnsys.2025.1687354

**Published:** 2026-04-29

**Authors:** S. Vidhusha, S. Karthika, N. Sahana, A. Sabari Srinivas, Shitharth Selvarajan, Nithya Rekha Sivakumar, Sumendra Yogarayan

**Affiliations:** 1 Immersive Technologies Lab, Department of Computer Science and Engineering, School of Engineering, Shiv Nadar University Chennai, Chengalpattu, India; 2 Department of Information Technology, Sri Sivasubramaniya Nadar College, Kalavakkam, India; 3 School of Built Environment, Engineering and Computing, Leeds Beckett University, Leeds, United Kingdom; 4 Department of Computer Science and Engineering, Chennai Institute of Technology, Chennai, India; 5 Centre for Research Impact and Outcome, Chitkara University Institute of Engineering and Technology, Chitkara University, Rajpura, Punjab, India; 6 Department of Computer Sciences, College of Computer and Information Sciences, Princess Nourah bint Abdulrahman University, Riyadh, Saudi Arabia; 7 Faculty of Information Science and Technology, Multimedia University, Melaka, Malaysia

**Keywords:** attention deficit hyperactivity disorder, deep learning algorithm, electroencephalography, learning disability, machine learning algorithms, remedials, statistical analysis

## Abstract

Learning disabilities in children are exhibited through difficulties in reading and writing due to lack of cognitive skills. It is generally diagnosed by analyzing the behavior and processing capacity of children by understanding their academic candidature. This can also be evidenced by capturing and analyzing their working memory patterns in the brain that show the effectiveness of therapeutic interventions in children with learning disabilities. This research works with the electroencephalography (EEG) signal data from the IEEE dataport consisting of 121 participants in total, of which 61 are ADHD and 60 are normal children aged 7–12 years. The usage of these data has influenced ground truth research by providing reliable data and mitigating the challenge with real-time availability of EEG data. This manuscript focuses on classifying the dataset into categories of children, viz normal and attention deficit hyperactivity disorder (ADHD), using brain connectivity parameters and validation through machine learning (ML) algorithms. Children with learning disabilities undergo therapeutic interventions to manage their disability. Generally, the progress of their intellectual capability can be assessed through visual cues and the responses that the children exhibit. Rather, their differences in brain cognition need to be analyzed to realize the outcomes of therapeutical effect. In this research, the brain connectivity parameters such as power spectral density (PSD), granger causality (GC), phase slope index (PSI), partial directed coherence (PDC), and directed transmission function (DTF) are estimated, quantified, and analyzed. Further, using the measures of brain connectivity parameters, certain ML algorithms—such as the logistic regression (LR), support vector machine (SVM), decision tree (DT), the k-nearest neighbor (KNN), and random forest (RF)—along with a deep learning model, viz. deep belief networks (DBN) have been employed for validating this study. Among these models, DBN offered a model accuracy of 89.7%. Hence, this concept emphasizes the validation and effectiveness of therapeutic interventions that can support clinical evaluations in children with learning disability.

## Introduction

**Figure 1:**
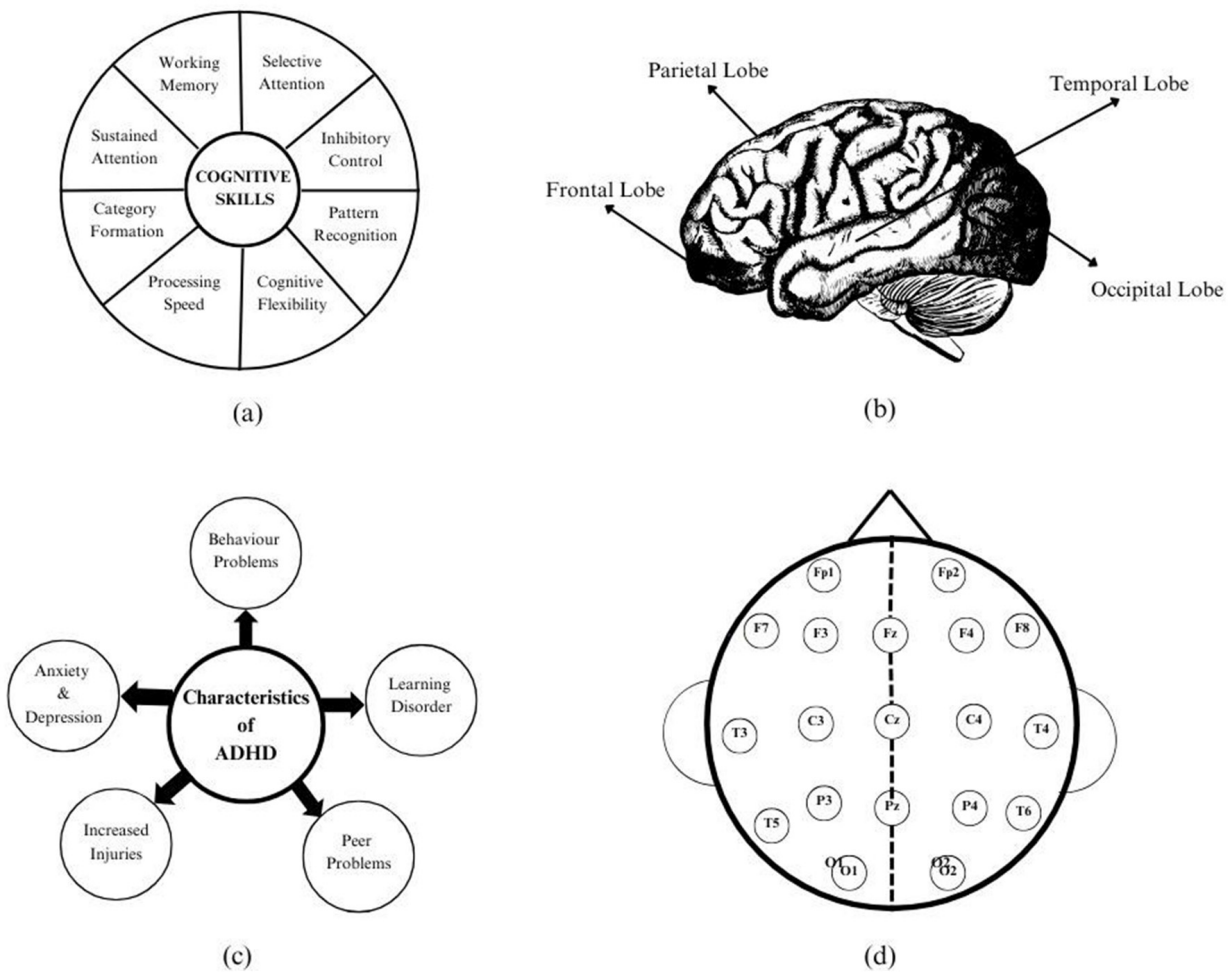
**(a)** Representation of cognitive skills. **(b)** Lobes of the brain. **(c)** Characteristics of ADHD. **(d)** Positioning of Emotiv electrodes.

Cognitive skills are a collection of abilities attained by efficiency of the brain lobes, as displayed in Figure 1a. As shown in Figure 1b, there are totally four lobes in the brain, namely frontal, parietal, occipital, and temporal. Frontal lobe is responsible for logical thinking and decision making, whereas the parietal lobe is responsible for sensory system processing, while the temporal lobe is responsible for auditory system processing, and the occipital lobe has the responsibility to process visual information. These lobes communicate and pass information in the form of electrical signals which is transmitted through millions of neurons available in the central nervous system (CNS).

Learning disability (LD) occurs due to the lack of one or more cognitive skills. Learning disabilities are of different types such as dyslexia, dyscalculia, dysgraphia, dyspraxia, etc., among which attention deficit hyperactivity disorder (ADHD) is one of the predominant learning disabilities. Children with ADHD face difficulty paying attention, leading to losing track of their work. Their characteristics are further discussed in Figure 1c. In therapeutic procedures, it is essential that the therapist must be aware of the child's initial level of learning disability to develop a treatment plan. Similarly, during the period of the treatment procedure, it is necessary to keep track of their level of learning to know whether the treatment has influenced them in managing their disability. Major improvements can be realized and assessed visually, but minor improvements that are not vocalized will ensure that the therapist continues treatment the same way for further progress to be measured using certain analytical techniques. However, assessing the impact of therapeutic procedures can be challenging for children with remedial needs, although brain improvements may occur in them, but the changes might not always be reflected in the child's behavior or characteristics.

Electroencephalography (EEG) signals are the recordings of electrical signals that a neuron transmits during communication between the lobes. The change in electrical activity is observed using EEG. Emotiv—a 19-channel device—is employed in which electrodes are placed on the scalp to record EEG signals. Electrodes—such as Fp1 and Fp2—correspond to the frontal-parietal region, while F7, F3, Fz, F4, and F8 are associated with the frontal region. T3, T5, T4, and T6 are in the temporal region, and C3, Cz, and C4 correspond to the central region of the brain. P3, Pz, P4, and P5 are placed in the parietal region, and O1 and O2 are placed over the occipital region of the brain, as shown in Figure 1d. The numbers in the electrodes do not have any specification. Working memory patterns are analyzed from the acquired EEG signals.

Therapeutic interventions are supported by analyzing and comparing the working memory pat terns of children with learning disabilities with those of normal children using statistical measures of the brain connectivity parameters. This approach emphasizes the application of methods to evaluate and validate the effectiveness of contribution therapeutic interventions in validating occupational therapy.

The concepts related to our approach followed by the methodology of our research—which consists of data collection with preprocessing phase, exploration on statistical parameter measures, and validation with ML algorithms and DL algorithm along with results and proof—are discussed in the following sections. Therefore, the main contribution of the research would be as follows:

- Analyze and provide custom-based assessment of brain connectivity parameters for individuals that can help therapists facilitate feature-specific treatment interventions.
- Estimate even minor improvements in brain connections for disordered children as an outcome of therapy, which currently may remain unvoiced due to the disabilities in the children.
- Figure out the optimal ML classifier model that can perform an accurate classification between disordered and normal children.

## Background and summary

Learning disabilities in children can be diagnosed and classified by interpreting the method through which the brain processes the information (Manghirmalani et al., 2011). Cognitive skills in children involve information processing and improve with a change in learning technique, and this can be observed using brain waves (Chrisilla et al., 2021). Children with learning disabilities find it difficult to pay attention and understand, when multiple items are placed for a task due to display factors, the child's attention becomes distracted, they find it difficult to stay focused on the task, and working memory is influenced by distraction, which can be diagnosed by recording and analyzing their working memory patterns from brain waves (Lenartowicz and Loo, 2014; Srinivasan et al., 2022; Mart'nez-Briones et al., 2021; Wang et al., 2022; Hollingworth and Beck, 2016; Hu et al., 2019; Frid and Manevitz, 2018; Mart'nez-Briones et al., 2020). Electroencephalography (EEG) signals that represent brain signals are recorded using brain computer interfaces (BCI) with electrodes placed in the brain area to help us acquire the working memory pattern that appears when the brain performs a task (Lenartowicz and Loo, 2014; Motie Nasrabadi et al., 2020; Zakopoulou and Miltiadous, 2022). Learning disabilities can be diagnosed by measuring the power distributed in the underlying frequency channels of EEG signals obtained by analyzing memory patterns. For example, ADHD factors are shown in EEG signals and are observed while measuring the band-wise power distribution (Mart'nez-Briones et al., 2021; Sandhya et al., 2015; Kuc et al., 2017; Mahmoodin et al., 2015; Khalaf et al., 2024). The influence of a channel over the other justifies the correlation between brain areas, where for children with LD there would be a poor influence, as the power of their channel frequency bands to solve the task would be low which can be statistically analyzed using brain connectivity parameters (Saleh, 2014). Learning disabilities progress according to age and other factors. Early detection of learning disability is a challenging task for which machine learning models are used; they can detect or diagnose disability by analyzing patterns from a few data samples through their training (Parmar and Paunwala, 2023; Seshadri N. P. G. et al., 2023; Seshadri N. P. et al., 2023; David and Balakrishnan, 2010; Chakraborty, 2020; Agrawal et al., 2024; Ahire et al., 2022, 2025; Tamboer et al., 2016; Atkar and Priyadarshini, 2020; Rezvani and Khorasani, 2019; Parmar et al., 2021). Machine learning models are used using EEG data to diagnose ADHD as an automated approach (Alim and Imtiaz, 2023). Machine learning techniques—such as multidimensional abnormalities, variational decomposition (VMD) with Hilbert transforms (HT), multi-resolutional analysis, and centrality measures—are used to classify children with ADHD and normal as an interpretable approach using EEG signals (Li et al., 2025; Khare and Acharya, 2023; Merudhula et al., 2024; Attallah, 2024). Furthermore, web-based PPS platforms have been developed for the earlier detection of ADHD using EEG data (Santarrosa-L'opez et al., 2025). As further advancement, deep learning models such as the graph convolutional network (GCN) are also used to extract multidomain features from EEG data to detect ADHD, and diagnostic interfaces supported with DL techniques are used to diagnose ADHD (Mao et al., 2025; Pappula and Anwar, 2024).

Various research studies that similarly support the diagnoses of learning disabilities have been performed specifically on dyslexia using machine learning techniques. However, this research focuses more on statistical analysis of brain connectivity parameters and incorporates state of-the-art ML/DL algorithms to understand the difference between ADHD and controls. Therefore, to realize the benefits of therapeutic interventions in ADHD children, the framework implemented in this investigation would be a pioneering approach to devise it.

## Methods

Therapeutic interventions are supported by analyzing and comparing the working memory patterns of children with learning disabilities with those of normal children using a statistical measure of the brain connectivity parameters. To quantify the improvement in their brain cognition, certain brain connectivity parameters are analyzed statistically and validated using algorithms such as SVM, LR, KNN, DT, RF, and DBN.

### Dataset description

The real-time data required for this research are highly comprehensive, and the availability of such data is limited. IEEE Dataport has positively influenced ground-truth research by providing reliable and well-structured data, thus mitigating the challenges associated with recording Electroencephalography (EEG) signals in real time.

**Figure 2:**
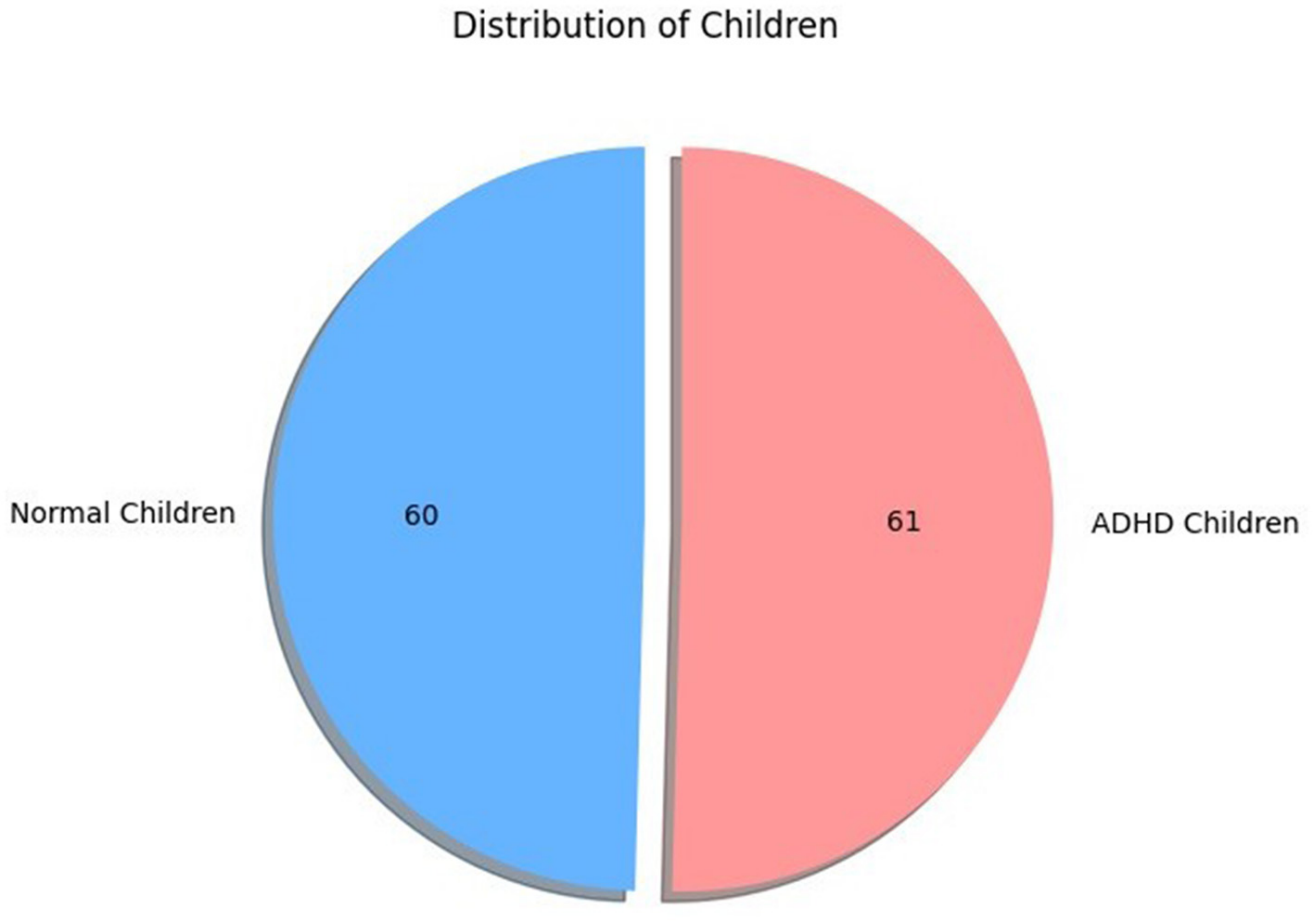
Dataset demographics: ADHD vs. normal children.

The EEG dataset is derived from the IEEE port, which is publicly available and 32 Mb in size. The dataset is categorized into ADHD and Normal children folders. The ADHD data contains two folders, and the Normal children contain two separate folders. There were 121 participants, of which 61 children diagnosed with ADHD and 60 normal children, demographically explained in Figure 2 with all aged between 7 and 12 years, as shown in Table 1. A psychiatrist diagnosed ADHD children according to the DSM-IV criteria, and signals were collected from children who consumed Ritalin for a period of 6 months for the IEEE EEG Dataset collected by Ali Motie Nasrabadi of Shahed University. Normal children were clear and had no history of psychiatric conditions or risk behaviors (Motie Nasrabadi et al., 2020). Their signals are recorded by assigning a visual attention task.

**Table 1:** Summary of dataset.

Parameters	Values
Number of ADHD children	61
Number of control children	60
Total number of participants	121
Sampling frequency	128 Hz
Number of channels (features)	19

### Data collection and pre-processing

In this study, data collection involves collecting and preparing data. EEG signals are recorded by assigning a visual attention task to the children using 10–20 standard 19-channel Emotiv device at 128 Hz sampling frequency as a non-invasive approach. The children received a set of 5–16 cartoon images and were instructed to count the number of images provided per iteration (Motie Nasrabadi et al., 2020). While processing their memory to count the images, the neuron activity in the brain lobes that passes information via electrical signals was recorded using an Emotiv device in EEG signal format and was given as an open source EEG dataset.

The signals in different folders of the same category of the dataset are appended together before pre-processing the data. The raw data might be noisy, and therefore the quality of the signals might also be poor. The Butterworth filter is used to eliminate the noise from the raw EEG signals. Transfer function: The influence of the artifacts can be minimized by passing EEG signals through a filter.

**Figure 3:**
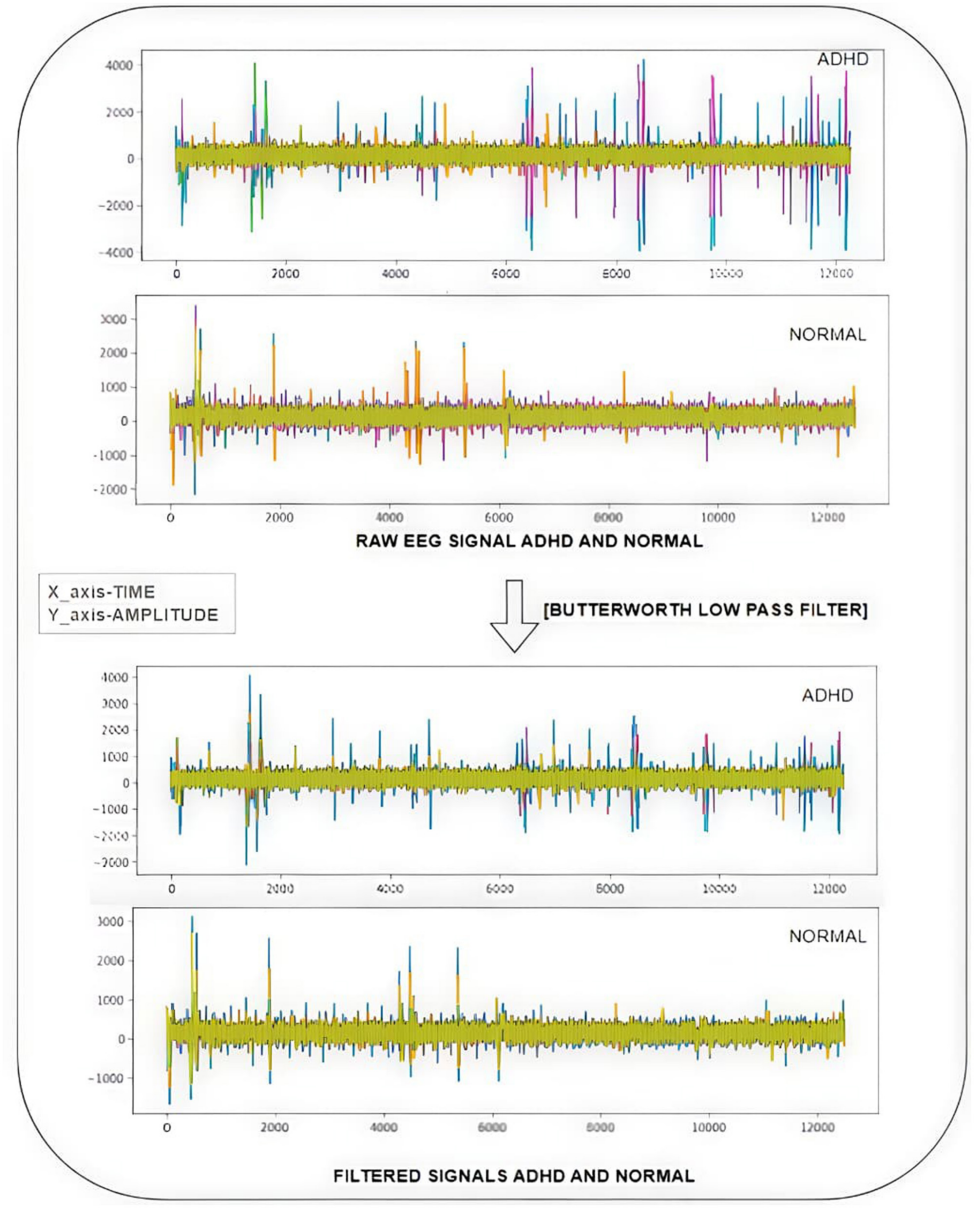
Workflow diagram for EEG signal preprocessing.

As the data are driven from IEEE Dataport, the information regarding the recording environment is not mentioned. So, general measures are taken to handle unwanted noise and artifacts as shown in Figure 3. The filter transfer function in the z-domain determines the mathematical connection among the input and output signals.


$$\text{H}(\text{z})\text{=}\frac{\text{f}(\text{z})}{\text{g}(\text{z})}\text{=}\frac{{\text{u}}_{0}\text{+ }{\text{u}}_{1}{\text{z}}^{-1}\text{ + }{\text{u}}_{2}{\text{z}}^{-2}}{1\text{ + }{\text{v}}_{1}{\text{z}}^{-1}\text{+ }{\text{v}}_{2}\;{\text{z}}^{-2}}$$ (1)

where

H(z) is the filter transfer function.

V(z) is the Z-transform of the output signal. U(z) is the Z-transform of the input signal.

u_0_ is the weight of current signal as input u(n).

u_1_z^−1^ is weight of input signal u(n-1) with one-step delay in time.

u_2_z^−2^ is weight of input signal u(n-2) with two-step delay in time.

Butterworth filter is a signal processing filter designed to provide a flat frequency response in the passband. The passband is the range of frequencies that it allows passing through the filter. This filter is broadly categorized into a low-pass Butterworth filter and a high-pass Butterworth filter. The raw EEG signals might have artifacts, and this affects the signal quality quite a lot. To remove this artifacts, a Butterworth filter is used to remove the noise. A low-pass Butterworth filter is especially chosen, as it can pass low-frequency signals such as the activity of the brainwave in the EEG data and significantly reduce high-frequency noise. Regardless of signaling morality, the signal acquisition process bound to have artifacts; due to children cooperation, and the signal is filtered to be uniform for customary phase, a Butterworth filter is used for the data.

A high-pass Butterworth filter allows signals with frequencies greater than the cut-off frequency to pass through the filter and attenuate signals with frequencies below the cut-off frequency. The cut-off frequency is the frequency at which the signals are attenuated if the signal frequency is below for the high-pass filter and above in the case of the low-pass filter than the cut-off frequency. The low-pass Butterworth filter is passed to the signals to attenuate high-frequency noise and muscle artifact, which will have a maximally flat frequency response. It attenuates the signal with frequency above the cut-off frequency and preserves the signal frequency below the cut-off frequency. The equation for low pass and high pass filter is given as Equation 1, whereas for low-pass filter, the coefficients will be very large positive numbers and for high-pass filter it will be alternating signs, where b and a are the normalization coefficients of cut-off frequency. Normalization of cut-off frequency is an important step in designing a Butterworth filter, as it ensures the performance accuracy of the filter.


$$\begin{array}{l} Normalization\;Cutoff\;Frequency=\frac{Cut-off\;Frequency}{Nyquist\;Frequency}\\ \;=0.4687 \end{array}$$ (2)

Nyquist frequency is the sampling frequency which helps to normalize the cut-off frequency. The order determines the steepness of the filter. The number of samples taken per second is the sampling frequency during the conversion of a continuous signal to a discrete signal.


$$\begin{array}{l} Nyquist\;Frequency=\frac{Sampling\;Frequency\;(f_{s})}{Order}=64 \end{array}$$ (3)

### Power spectral density (PSD)

PSD is calculated to measure how much power is distributed in each frequency band of the signal. This analysis is derived from the concept of discrete Fourier transform (DFT).


$$\begin{array}{l} \text{U}\left(\text{k}\right)=\overset{{\text{N}}_{\text{p}}-1}{\underset{\text{n}=0}{\sum }}\text{u}\left[\text{n}\right]{\text{e}}^{\frac{-\text{j}2\pi \text{kn}}{{\text{N}}_{\text{p}}}} \end{array}$$ (4)

Here, u[n] is discrete with U(k) as the DFT. N_p_ symbolizes the number of points in DFT, and k is the frequency ind


$$\begin{array}{l} \text{Power Spectral Density},\text{PSD}\left(\text{k}\right)=\frac{\text{PU}[\text{k}]}{\text{Δfreq}} \end{array}$$ (5)

Delta, Theta, Alpha, Beta, and Gamma are the frequencies collectively associated with each EEG frequency band. These bands are individually responsible for specific functions, as shown in Table 2. Based on the power distribution in each band, the ability of the brain can be measured, i.e., normal children will have good range of Delta, Theta, and Beta compared to the children with learning disability while performing a task. So, for the visual attention task, Delta, Theta, Alpha, Beta band measures are accounted through this technique, and two classes of children can be classified.

**Table 2:** Bands and responsibilities.

Frequency bands	Range (Hz)	Responsible states
Delta	0.1–4	Attention (rest/awake)
Theta	4–8	Memory and learning
Alpha	8–14	Cognitive alertness
Beta	14–32	Cognitive processing
Gamma	32–100	Observation and active response

Band spectrum analysis also helps segment a signal into independent frequencies to indicate the significance of the band power.

### Statistical analysis

**Figure 4:**
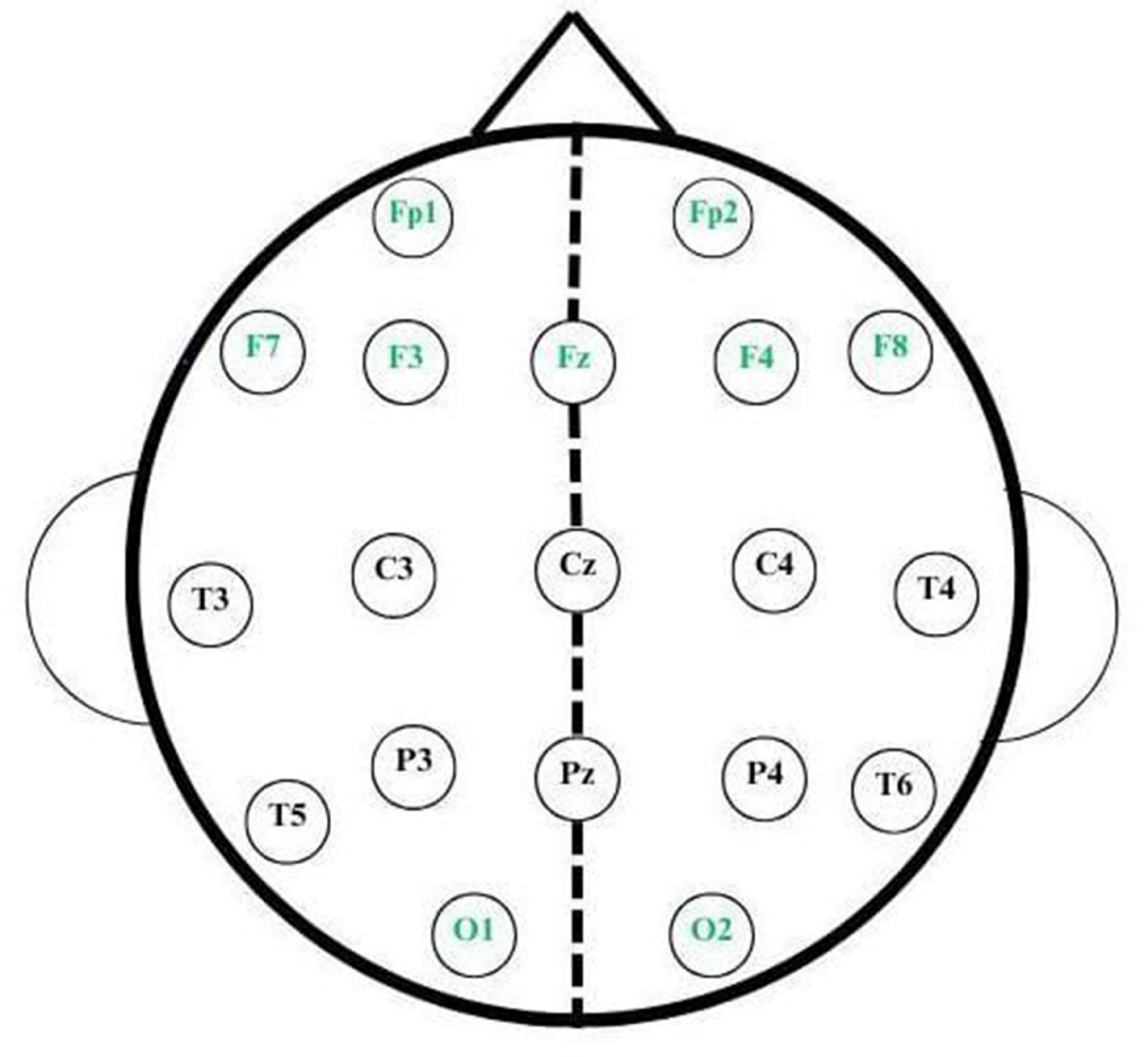
Representation of frontal and occipital lobes monitoring channels are the active channels indicated in green.

On the dataset, various statistical parameters are analyzed and visualized using tools such as coherence, granger causality, PSI, PDC, DTF, and others; the efficiency of connectivity among the brain's frontal and occipital lobes can be inferred with the help of these parameters, and those lobes are important because they play a major role in visual attention task, as the frontal lobe helps the child count the number of cartoon images in association with the occipital as it contributes by communicating the visual images information to the brain, i.e., the corresponding channels such as Fp1, Fp2, F3, F4, F7, F8, Fz, O1, and O2 signals are measured as mentioned in Figure 4. The Alpha band of these channels are statistically analyzed to classify and find the difference between children with learning disability and normal children because Alpha band is more associated for processing visual attention task, as shown in Table 2.

### Coherence

Coherence quantifies the strength in association of two regions of the brain that focus on performing actions such as learning, responding, eating, etc. The coherence is represented as a matrix. A coherence matrix is the mathematical representation that provides the coherence between multiple regions. In the EEG dataset, the coherence matrix represents the linear dependency between the brain regions by comparing the signals acquired from each channel. The range of coherence is between 0 and 1, for a high coherence between two bands, it is interpreted by a value close to 1 and for a low coherence between two bands, it is interpreted by the value close to zero. The diagonal elements represent coherence with itself, and thus it is always 1. The effectiveness of the associative relationship between the frontal and occipital lobes is observed using the representation of the coherence matrix.

### Granger causality

Granger causality is a statistical measure that determines whether a time-series-based signal can predict the future value of another time-series-based signal. This measure helps to understand the direct influence of a brain region on another region, which means that it depicts how well an activity performed at one region of the brain affects the other region of the brain. This aims to understand and perform specific cognitive skills and helps to identify the direction through which information flows among the brain regions. The range of granger causality varies from 0 to ∞. The lower the values, the lower the influence of the region over the other, and vice versa (Saleh, 2014). The value of transfer function H_uu_(freq) that represents the influence of channel *v* on channel *u* must not be zero to analyze the causal difference. If the transfer function is zero then no causal difference between channels will be observed. Here, granger causality is measured to ensure the influence of the occipital lobe on the frontal lobe and the flow of visual information to count the number of images. It is also represented as a matrix to display the effect of causality in frontal by occipital.


$${\text{F}}_{\text{v}\to \text{u}}\left(\text{freq}\right)=\text{ln}(\frac{{\text{S}}_{\text{uu}}\left(\text{freq}\right)}{|{\text{H}}_{\text{uu}}(\text{freq}){|}^{2}{\text{Σ}}_{\text{uu}})}$$

S_uu_(freq) is the PSD of channel u, H_uu_ is the transfer function from *v* to *u*, and Σ_uu_ is the noise variance channel u.

### Phase slope index (PSI)

To estimate both the magnitude and the direction of flow of information in the EEG dataset, PSI involves fast Fourier transform (FFT) method. It helps in measuring the temporal order between spatially separated signals, simply getting the phase linearity among two signals. The PSI is calculated as:


$$\begin{array}{l} {\text{Ψ}}_{\text{vu}}=\text{τ}\left[\underset{\text{freq}\text{F}}{\sum }{\text{C}}_{\text{vu}}*\left(\text{freq}\right){\text{C}}_{\text{vu}}\left(\text{freq}+\text{Δfreq}\right)\right] \end{array}$$ (6)

where Ψ_uv_ is the function of coherence between channel u and v, the frequency resolution is given as Δfreq, and the imaginary part of coherency is given as I. The coherency is defined as:


$$\begin{array}{l} {\text{C}}_{\text{vu}}\left(\text{freq}\right)=\frac{{\text{S}}_{\text{vu}}\left(\text{freq}\right)}{\sqrt{{\text{S}}_{\text{vv}}\left(\text{freq}\right)\cdot {\text{S}}_{\text{uu}}\left(\text{freq}\right)}} \end{array}$$ (7)

Where

C_vu_(freq) is the coherency between *v* and *u* at a frequency *freq*.

S_uu_(freq) is the PSD of the u channel at frequency *freq*.

S_vv_(freq) is the PSD of the v channel at frequency *freq*.

S_vu_(freq) is the cross-spectral density between channels v and u at frequency freq.

The imaginary part symbolizes the role of the channels, that is, if Ψ_vu_ > 0 the channel with signal v drives and the channel with signal u responds to it. If Ψ_vu_ < 0 channel with signal u drives channel with signal v, i.e., –Ψ_vu_ the information flows in opposite direction. PSI is measured to know the trace of direction in which the information flows among frontal and occipital lobes by analyzing the phase relationship between signals of the corresponding channels which monitor the lobes.

### Partial directed coherence (PDC)

PDC measures the flow of information intensity among the brain regions in the EEG data using a granger causality measure taken in the frequency domain. It is a direct connectivity that takes the measure of inverse of transfer function involved in granger causality. In this technique, directed functional connectivity is measured between regions of the brain. Quantifies the influence and strength of the connection between two regions. The range of PDC is between 0 and 1. If the values are closer to or equal to zero, it indicates that the influence of the brain region which channel j is monitoring over the region of the brain channel i handing is less. Similarly, if the values are closer or equal to 1, then the influence of brain region which channel v is handling is greater over the region channel u is responsible, that is, the strength of the connection and the influence capacity from occipital to frontal is analyzed to differentiate children with learning disabilities and normal children.


$${\text{PDC}}_{\text{vu}}(\text{freq})\text{=}\frac{\left|{\overset{-}{\text{H}}}_{\text{uu}}(\text{freq})\right|}{\sqrt{\sum_{\text{m=1}}^{\text{i}}{\left|{\overset{-}{\text{H}}}_{\text{um}}(\text{freq})\right|}^{\text{2}}}}$$ (8)

where PDC_vu_(freq) is the partial directed coherence, −H_*uv*_ (freq) is the inverse transfer function that indicates the influence of a brain region channel v is handling over the region channel u at a frequency freq. The summation m = 1 represents the normalization of the influence in all channels.

### Directed transmission function (DTF)

DTF determines the strength of a linear relationship between signals and the directionality of the relationship, and focuses on how much signal power from a brain region is transferred to another region which is monitored via channels at a particular frequency, i.e., occipital channels to frontal channels. It enables the construction of directed brain networks, providing insights into functional and structural connectivity. It is derived from granger causality and multi-vector autoregressive model (MVAR), given the transfer matrix of system H(f).

Granger causality,


$${\text{F}}_{\text{v}\to \text{u}}\left(\text{freq}\right)=\text{ln}\frac{{\text{S}}_{\text{uu}}\left(\text{freq}\right)}{{\text{S}}_{\text{uu}}\left(\text{freq}\right)-|{\text{H}}_{\text{vu}}\left(\text{freq}\right){|}^{2}{\text{Σ}}_{\text{vv}}}$$

MVAR model,


$$\text{U}\left(\text{t}\right)=\sum_{\text{i}=1}^{\text{o}}{\left(\text{Coef}{\text{f}}_{\text{A}}\right)}_{\text{k}}\text{U}\left(\text{t}-\text{i}\right)+\text{N}\left(\text{t}\right)$$

where U(t) is the MVAR model vector of signal for all signals at t with Coeff_A_ as the coefficient of autoregressive “o” as the order of the model with “N” the vector of noise at time t.

The relation among the EEG data channels can be analyzed with the DTF information, including the phase relations between signals. The Directed Transfer function is given as


$$\begin{array}{l} {\left(\text{DTF}\right)}_{\text{v}\to \text{u}}\left(\text{freq}\right)=\frac{|{\text{H}}_{\text{uu}}\left(\text{freq}\right){|}^{2}}{\sum_{\text{m}=1}^{\text{i}}|{\text{H}}_{\text{um}}\left(\text{freq}\right){|}^{2}} \end{array}$$ (9)

The above equation is the normalized version of DTF, whose input range is between 0 and 1 and produces a relationship between the inflow from the channel v region to the channel u region and all the inflows to the channel u region. The DTF describes at frequency freq the influence of channel u's region on channel v's region with m as the index for all channels.

### Validation using machine learning algorithms

ML techniques are employed to validate the measures that we found using the analysis of brain parameter data, the EEG signal. The data is pre-processed in such a way as to train a particular algorithm of the model in order to predict and classify the results. There are two types of ML models: classification and regression. To validate, we make use of classification ML algorithms like SVM, LR, RF, KNN, and DT.

True positives (TP), true negatives (TN), false positives (FP), and false negatives (FN) instances are represented in the heat map form so-called “confusion matrix” upon which the classification metrics are used. As a result of the model's working, the accuracy of the model's classification is calculated as the proportion of absolutely correct predictions out of all predictions made by the model. Estimation of the model's performance on different subset of dataset, i.e., splits of the dataset can be computed using cross-validation scores, thus helping to evaluate the quality and generalizability of algorithms on various sets of the data.

The data for both ADHD and control groups are loaded from CSV files. Each dataset contains PSD features (Alpha, Beta, Gamma, and Theta bands) that represent the brainwave communicating activity of the children in each group. The label column (“Label”) is added to each dataset, where 1 represents ADHD and 0 represents control, marking the target for classification. The datasets are then concatenated into a single dataset, combining the features of both groups. The relevant features for classification, which are the frequency bands, are selected for the model input. These features are assumed to capture distinct brain activity patterns that can differentiate ADHD and normal children.

### Logistic regression (LR)

It is a ML algorithm of supervised learning, widely used for two class classification. LR is categorized as multinomial LR and ordinal LR. It assumes linear relationship among the independent features and the logits of the dependent variable. LR is a linear model that measures the occurrence of a binary outcome (ADHD or control) based on input characteristics. LR works by identifying a decision boundary that best separates the both the classes, such that minimizes the binary cross-entropy loss. It uses sigmoid function, a mathematical function whose curve is in an “S” shape and is used to map the predicted values to probabilities. Sigmoid function takes the independent features and produces a probability value between 0 and 1.

The standard scaler is given by,


$$\begin{array}{l} {\text{X}}_{\text{new}}=\frac{{\text{X}}_{\text{i}}-{\text{X}}_{\text{mean}}}{\sigma } \end{array}$$ (10)

where σ is the standard deviation.

For a given input X = {x_1_, x_2_, x_3_,…, x_i_}, apply the multilinear function to the input x:


$$\begin{array}{l} \text{z}=\text{WX}+\text{b} \end{array}$$ (11)

where W is the vector of weight with bias as b, and then apply the sigmoid function, the algorithm used for the logistic regression is discussed in Algorithm 1.

Algorithm 1 Logistic regression with hyperparameter tuning using GridSearchCV on processed EEG signal dataset.

**Require:** Processed EEG Signal dataset {X, y}, train-test split ratio, regularization strength C, optimization algorithm “solver”, number of iterations “max” iterBestlogisticregressionmodelβ^*^
**Ensure 1:** Normalize and preprocess the dataset to get X_s*caled*_
2: 80-20 Dataset split applied on X_s*caled*_ for training and testing
3: Define hyperparameter search space:
    • C *arrow* {0.1, 1, 10} *(Regularization strength)*
    • solver *arrow* {lbfgs, liblinear, saga} *(Optimization algorithm)*
    • max_iter *arrow* {100, 200, 300} *(Number of iterations for convergence)*
4: Initialize GridSearchCV (k-fold cross-validation with k = 5):
    • Instantiate GridSearchCV object:
    • grid_search *arrow* GridSearchCV(LogisticRegression (random_state = 42)),
    • Train the logistic regression model with hyperparameter tuning:
    • grid_search.fit(X_t*rain*_, y_t*rain*_)
    • Get the best model after tuning:  •β^*^arrow grid_search.best_estimator_
    • Predict on the validation set:
    • y_p*red*_ *arrow* β*(X_v*al*_)

### K-nearest neighbor (KNN)

This is a supervised ML algorithm which is non-parametric that is used for regression and classification tasks. It is based on proximity between the data points to predict or classify for grouping the data points. The KNN algorithm identifies the nearest neighbors for the given datapoint based on which a class label is assigned. The nearest neighbors are identified by calculating how close they are from the data point for which the distance metrics. The distance between data points are measured by Euclidean distance given as,


$$\begin{array}{l} \text{d}\left(\text{x},\text{y}\right)=\sqrt{\overset{\text{n}}{\underset{\text{i}=1}{\sum }}}{\left({\text{y}}_{\text{i}}-{\text{x}}_{\text{i}}\right)}^{2} \end{array}$$ (12)

The Manhattan distance is also widely used to measure distance between two points by calculating the actual difference among the values of two data points. The Minkowski distance is like a generalized version of distance metrics like Euclidean, etc. The Hamming distance is used for Boolean or string vectors, identifying the points where the vectors do not match. Euclidean distance metric is used in the algorithm for this study which maintains the geometric relationship between the datapoints. The formal representation of the methodology used for the KNN is given in Algorithm 2.

Algorithm 2 KNN classifier with hyperparameter optimization using GridSearchCV on EEG signal dataset.

**Require:** EEG Signal dataset {X, y}, train-test split ratio, number of neighbors n, weighting function, and distance metric
**Ensure:** Best KNN model β^*^
1: Normalize and preprocess the dataset to get X_s*caled*_ 2: 80-20 Dataset split applied on X_s*caled*_ for training and testing 3: Define the hyperparameter search space:
    • n_neighbors *arrow* {3, 5, 7, 9} *(Number of neighbors)*
    • Weights *arrow* {uniform, distance} *(Weighting function)*
    • Metric *arrow* {euclidean, manhattan, minkowski} *(Distance metric)*
4: Initialize GridSearchCV (k-fold cross-validation with k=5):
    • Initialize GridSearchCV:
    • grid_search *arrow* GridSearchCV (KNeighborsClassifier(),
    • Train the KNN model:
    • grid_search.fit(X_t*rain*_, y_t*rain*_)
    • Obtain the best model:  •β^*^arrow grid_search.best_estimator_
    • Predict on the validation set:
    • y_p*red*_ *arrow* β*(X_v*al*_)

#### Support vector machine (SVM)

This works well on small and complex datasets. The ideology behind SVM is find a best fit decision boundary, also called as hyperplane that can separate and add n-dimensional data points into the classes. Support vectors of SVM fits the best hyperplane that are the extreme points that are nearer to the hyperplane which determines the hyperplane. Margin is the distance between hyperplane and support vector, and if the margin is large then it is a good fit of model as it will maximize the decision boundary among classes. The margin is of two categories: soft margin and hard margin. Hard margin finds a boundary that completely divides the data points belonging to different classes, such that it ensures maximum width possible. The equation of hyperplane to be optimized when the margin is hard is given below,


$$\begin{array}{l} \text{arg max}\left({\text{w}}^{\to }\text{*,b*}\right)\frac{\text{2}}{\text{||w||}}{\text{such that y}}_{\text{i}}{\text{(w}}^{\to }{\text{·x}}^{\to }{\text{+b}}^{\to }\text{)}\geq \text{1} \end{array}$$ (13)

The soft margin fits a hyperplane in such a way that it allows some misclassification or margin violation. This is more suitable when anomaly is present in the data. The equation of hyperplane that is to be optimized is given as,


$$\begin{array}{l} \text{arg}\begin{array}{c} \min\\ w^{*},b^{*} \end{array}\frac{||\text{w}||}{2}+\text{c}\cdot \overset{\text{n}}{\underset{\text{i}=1}{\sum }}{\text{C}}_{\text{i}} \end{array}$$ (14)

There are two types of SVM: linear (used when data are perfectly linear) and non-linear SVMs (used when data are non-linear). Non-linear SVMs are used to capture the non-linearity in the “Kernel Trick.” The kernel trick maps low dimensional space to high-dimensional space using mathematical functions, also called kernels. There are multiple types of kernels,

Linear kernel,


$$\begin{array}{l} \text{k}\left(\text{u},{\text{u}}^{,}\right)={\text{u}}^{\text{T}}{\text{u}}^{,} \end{array}$$ (15)

Polynomial kernel,


$$\begin{array}{l} \text{f}\left({\text{U}}_{1},{\text{U}}_{2}\right)={\left({\text{U}}_{1}^{\text{T}}{\text{U}}_{2}+1\right)}^{\text{d}} \end{array}$$ (16)

where U1 and U2 are feature vectors, and d is the degree of the polynomial sigmoid function,


$$\begin{array}{l} f\left({\text{u}}_{1},{\text{u}}_{2}\right)=\text{tanh}\left(\alpha \cdot {\text{u}}^{T}\text{v}+\text{u}\right) \end{array}$$ (17)

RBF kernel maps the low dimensional data points to a high dimensional space with a non-linear function.


$$\begin{array}{l} f\left({\text{u}}_{1},{\text{u}}_{2}\right)={\text{e}}^{-\frac{||{\text{u}}_{1}-{\text{u}}_{2}{||}^{2}}{2\sigma^{2}}} \end{array}$$ (18)

In this study, linear kernel is chosen to employ in the SVM model which helps to interpret the dataset by reducing the complexity through overfitting. The algorithm used for the SVM is given in Algorithm 3.

Algorithm 3 SVM classifier with hyperparameter tuning using GridSearchCV on EEG signal dataset.

**Require:** EEG Signal dataset {X, y}, train-test split ratio, regularization parameter C, kernel
type, and gamma coefficient
**Ensure:** Best SVM model β^*^
1: Normalize and preprocess the dataset to obtain X_s*caled*_
2: 80-20 Dataset split applied on X_s*caled*_ for training and testing
3: Define hyperparameter search space:
    • C *arrow* {0.1, 1, 10} *(Strength of regularization)*
    • Kernel *arrow* {linear, rbf, poly} *(Type of kernel)*
    • Gamma *arrow* {scale, auto} *(Coefficient of kernel)*
4: Set up GridSearchCV (k-fold cross-validation with k=5):
    • Create GridSearchCV:
    • grid_search *arrow* GridSearchCV(SVC(),
    • {C : C, kernel : kernel, gamma : gamma},
    • Train the SVM model:
    • grid_search.fit(X_t*rain*_, y_t*rain*_)
    • Get the best model:  •β^*^arrow grid_search.best_estimator
    • Predict on the validation set:
    • y_p*red*_ *arrow* β*(X_v*al*_)

#### Decision tree classifier (DT)

It is applied very extensively to classification as well as regression problems in supervised learning. Dataset is repeatedly divided into subsets based on feature values to construct a model, where every internal node is a decision-made node over a feature, and every edge node is the result of the decision, and every leaf node is a terminal class label or output. The aim is to construct a model which forecasts the value of a target variable by discovering straightforward decision rules induced from the features of the data. Interpretability is one of the significant strengths of decision trees; rules produced by the tree are easy to visualize and comprehend, making them valuable in explaining model choices. They also have the capability to deal with both numerical and categorical data and need minimal preprocessing of data. Nonetheless, decision trees have a number of drawbacks, such as overfitting the training data, thus being less generalizable to new data. They are also prone to small changes in the data, which can cause instability in the model structure. In addition, they can be biased toward features with higher levels and can fail to capture complex interactions between features. These shortcomings can be overcome using ensemble techniques, i.e., RF and Gradient Boosting to merge several decision trees to achieve higher accuracy and generalization.


$$\begin{array}{l} \text{Gini}=1-\overset{{\text{N}}_{\text{c}}}{\underset{\text{u}=1}{\sum }}\text{p}{\text{i}}^{2} \end{array}$$ (19)

where N_c_ is the number of classes, and p_i_ is the proportion of a specific class u at a node.


$$\begin{array}{l} \text{Entropy}=-\overset{\text{N}}{\underset{\text{u}=1}{\sum }}{\text{p}}_{\text{i}}\text{lo}{\text{g}}_{2}\left({\text{p}}_{\text{i}}\right) \end{array}$$ (20)

Here, p_i_ is the probability of class u.


$$\begin{array}{l} \text{IG}=\text{Entropy}\left(\text{parent}\right)-\overset{\text{k}}{\underset{\text{v}=1}{\sum }}\frac{{\text{n}}_{\text{j}}}{\text{n}}\cdot \text{Entropy}\left(\text{j}\right) \end{array}$$ (21)

where n_v_ is the count of sample in class v, and n is the total count of samples. The pseudocode used for the Decision Tree is presented in Algorithm 4.

Algorithm 4 Decision tree classifier with hyperparameter optimization using GridSearchCV on EEG signal dataset.

**Require:** EEG Signal datasets for ADHD and Control, feature list {X, y}, train-test split ratio, hyperparameter grid
**Ensure:** Best Decision Tree model θ^*^
1: Normalize and preprocess the dataset to get X_s*caled*_
2: 80-20 Dataset split applied on X_s*caled*_ for training and testing
3: Define hyperparameter search space:
    • Criterion: {gini, entropy, log_loss}
    • Splitter strategy: {best, random}
    • Maximum tree depth: {None, 5, 10, 20, 50, 100}
    • Minimum samples for node split: {2, 5, 10}
    • Minimum samples per leaf: {1, 2, 4}
    • Feature selection strategy: {None, sqrt, log2}
    • Class weight scheme: {None, balanced}
4: Train and evaluate Decision Tree using GridSearchCV:
    • Setup GridSearchCV (k-fold cross-validation with k=5)
    • Fit the model on training data: grid_search.fit(X_t*rain*_, y_t*rain*_)
    • Extract best estimator: θ^*^arrow grid_search.best_estimator_
    • Predict on validation set: y_p*red*_ *arrow* θ^*^(X_v*al*_)
    • Compute evaluation metrics: classification report, confusion matrix,
       and accuracy
    • Compute 5-fold cross-validation score using θ^*^

#### Random forest classifier (RF)

This is a ML algorithm for supervised learning that is widely employed for classifying tasks and is based on decision trees. Decision trees is a classification ML algorithm that works based on a set of rules that separates the heterogeneous population of data points into homogeneous subgroups. Ensemble techniques make use of combination of models to increase accuracy. There are multiple types of ensemble techniques of decision tree, such as bagging, which the average of the predictions made by multiple classifier models is taken as the ultimate prediction. Boosting is a technique in which the weighted vote of the predictions of multiple classifiers is taken for the closing prediction. RF is an ensemble technique where multiple classifiers are trained on different subsamples of the data. This subsample of data is given by the Bootstrap technique where multiple subsamples of the data are taken, which resembles the same property as the original dataset, and it uses sampling with replacement. In every subsample, a decision tree is trained with data, and in classification the majority votes are considered among multiple classifiers of random forest. RF classifier randomly selecting subsets of features and trains trees on those subsets to reduce overfitting and increase model robustness. The formal representation of the Random Forest model is given in Algorithm 5.

Algorithm 5 Random forest classifier with hyperparameter optimization using GridSearchCV on EEG signal dataset.

**Require:** EEG Signal dataset {X, y}, train-test split ratio, number of trees n, min samples leaf, min samples split, bootstrap option and max depth.
**Ensure:** Best Random Forest model β^*^
1: Normalize and preprocess the dataset to get X_s*caled*_
2: 80-20 Dataset split applied on X_s*caled*_ for training and testing
3: Define the hyperparameter search space:
    • n_estimators *arrow* {50, 100, 200} *(Number count of trees )*
    • Max depth *arrow* {None, 10, 20, 30} *(Count of max tree depth)*
    • Min samples split *arrow* {2, 5, 10} *(Minimum no. of samples to split)*
    • Min samples leaf *arrow* {1, 2, 4} *(Minimum no. of samples at leaf)*
    • Bootstrap *arrow* {True, False} *(Bootstrap sampling)*
4: Set up GridSearchCV (k-fold cross-validation with k=5):
    • Train the Random Forest model:
    • grid_search.fit(X_t*rain*_, y_t*rain*_)
    • Get the best model:  •β^*^arrow grid_search.best_estimator_
    • Obtain prediction on validation set:
y_p*red*_ *arrow* β*(X_v*al*_)

### Validation using deep learning algorithm

It is a subgroup of ML with deep network layers used for complex analysis and processing of complex data which helps to capture the temporal. Hence, they work similar to ML model but involves layers of cells with deep networks. Deep learning algorithm such as DBN is used to validate the classification of children with ADHD and normal children by analyzing band-wise power distributed data for each signal of the children.

#### Deep belief network (DBN)

This is a DL algorithm which can be used for classification task for complex analysis. It consists of input layer, restricted Boltzman machines (RBM) layers as the hidden layers, and output layer.

The RBMs layer will analyze the input data, undergo feature selection, and understand the pat terns from the processing of multiple RBMs layer stacked deep in network which is an unsupervised pretraining. Features are extracted by hierarchical learning techniques of RBMs, through which frequency domain differences can be captured by understanding the relation between band frequen cies. Furthermore, in the output layer, classification is performed with help of activation functions such as ReLU, Sigmoid, etc. The discussed algorithm used for the DBN is formally represented as in Algorithm 6.

Algorithm 6 Deep Belief Network (DBN) training with Gaussian-Bernoulli RBMs and Fine- Tuned MLP. 
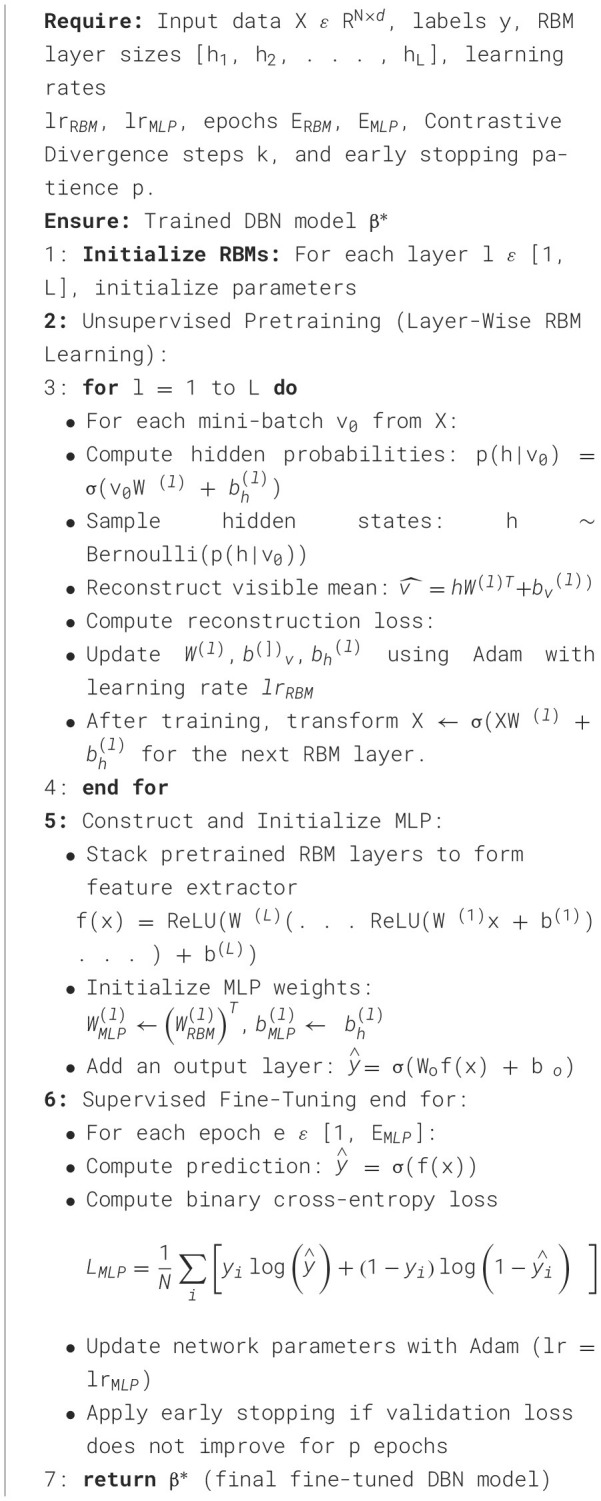

## Technical validation

The methods discussed in the previous sections are used, and the evaluation of the results with validation is inferred and explained in the following sections.

**Figure 5:**
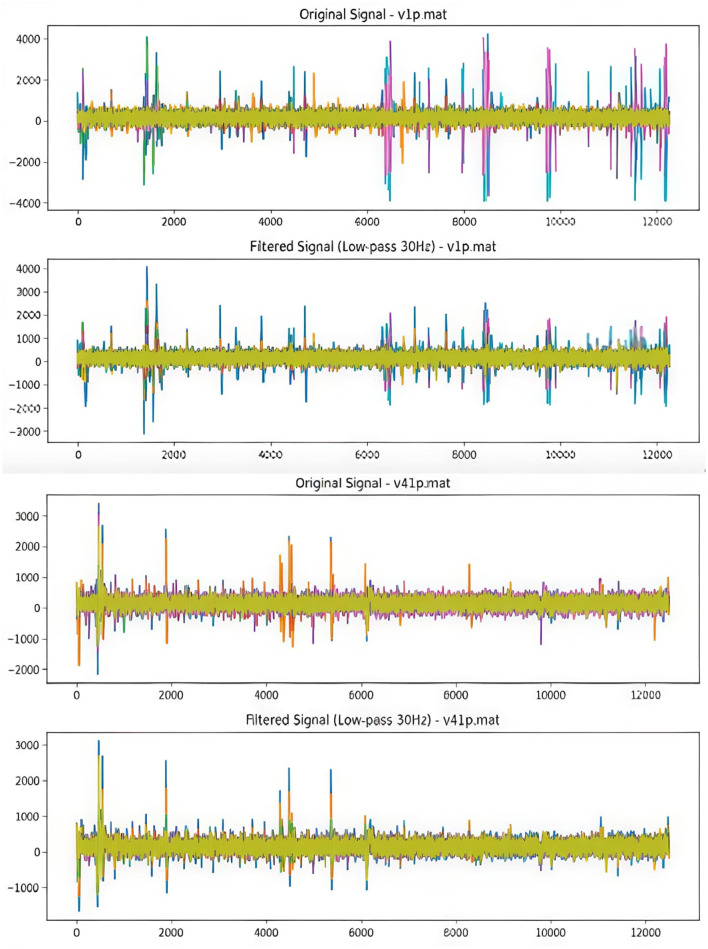
Using Butterworth filter raw EEG signals are filtered, respectively, and as an instance v1p.mat raw signal of an ADHD child and v41p.mat raw signal of a control child filteration process are illustrated.

As shown in Figure 5, the noise in the raw data is filtered using techniques such as transfer function, and low-pass Butterworth filter is applied in the raw EEG signals for ADHD and normal children. The sampling frequency (fs) is set to “128 Hz.” The order is equal to “2.” The cut-off frequency is set to 30 Hz.


$$\begin{array}{l} \text{Nyquist Frequency}=\frac{128}{2}=64 \end{array}$$ (22)

$$\begin{array}{l} \text{Normalization of Cut}-\text{off Frequency}=\frac{30}{64}=0.4687 \end{array}$$ (23)

Even though with maximum measurable frequency as 64 Hz, cutting-off frequencies above 30 Hz are because the visual attention task is a part of normal function activity which does not involve any complex cognition, gamma bands have been excluded. However, Alpha and Beta deal with cognitive alertness and processing while the child is at rest, as well as being involved in a task.

The high frequency might also be influenced by muscle artifacts or electrical noises, since the details regarding the data collection environment have not been disclosed by the dataset author in IEEE Dataport, for the purpose of maintaining standard input, this measure has been handled.

### PSD

**Figure 6:**
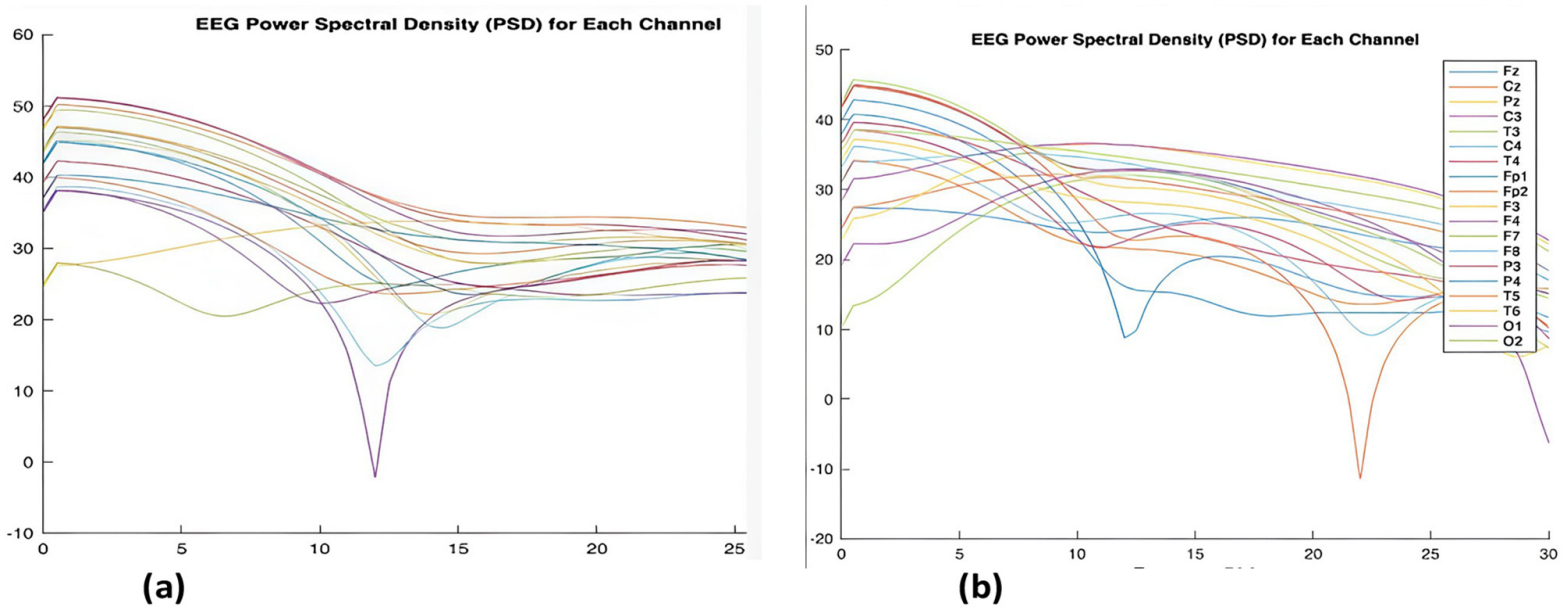
**(a)** Representation of EEG Power Spectral Distribution recorded through 19 channel from ADHD. **(b)** Representation of EEG Power Spectral Distribution recorded through 19 channel from Control.

Power spectral density is calculated for filtered signals. Figure 6a shows the PSD being applied for dataset of ADHD children, and Figure 6b shows that the PSD is applied for normal children. The analysis reveals that the child with learning disability exhibits higher Delta and Theta power, relatively lower Alpha and Beta power compared to the normal child, which indicates that the brain connectivity for learning and understanding in control children is higher than ADHD children.

#### Band spectrum analysis

**Figure 7:**
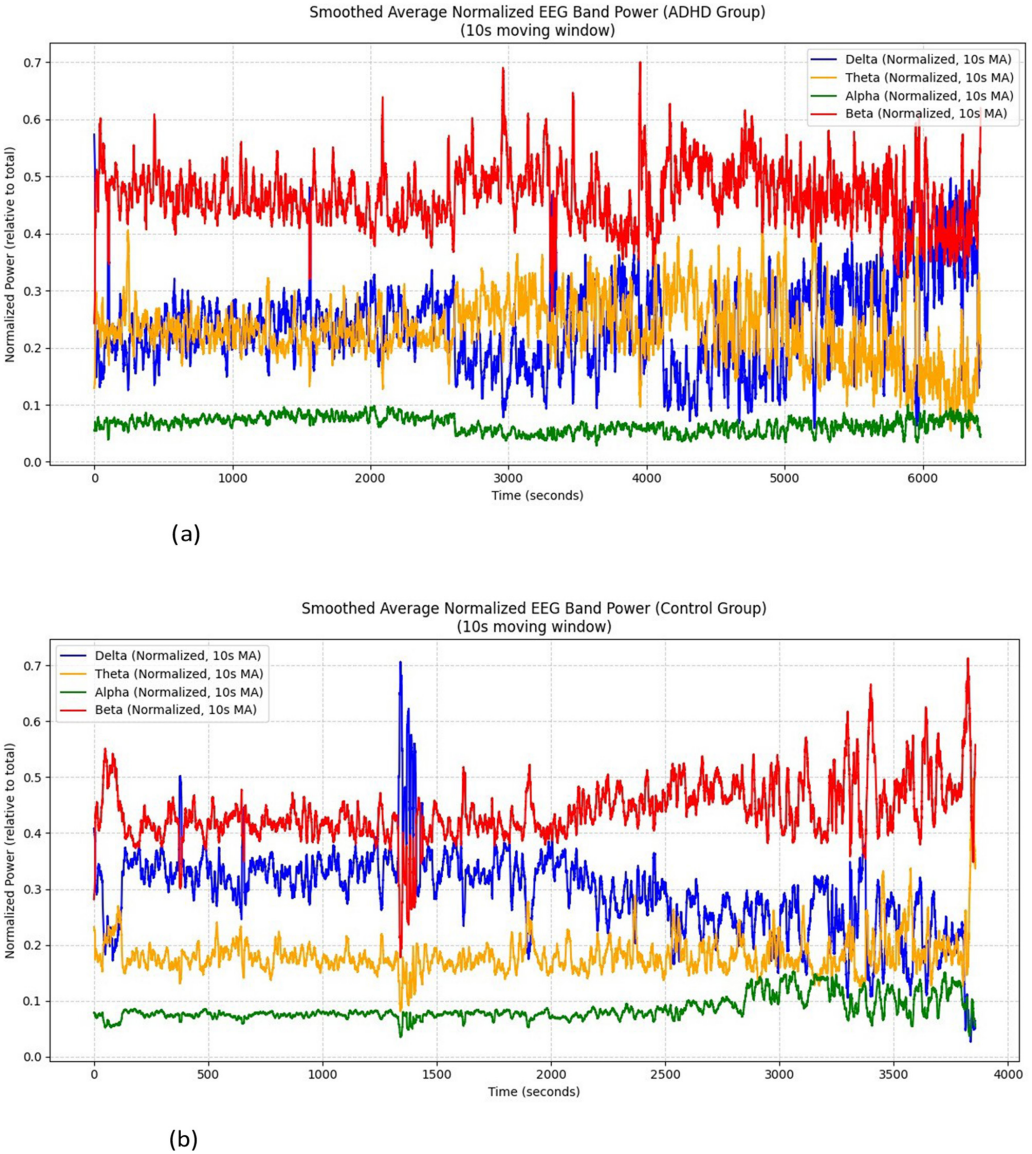
**(a)** Analysis of band spectrum across all 19 channels of ADHD group. **(b)** Analysis of band spectrum across all 19 channels of control group.

As shown in Figures 7a, b, the significance difference between the ADHD group data and the control group has been observed by analyzing their band power spectrum for each channel through a p-test, where the Beta power for cognitive processing is spiked with a combination of delta responsible for restfulness, aligned for change in rest to pay attention during visual attention task in the controls data effectively than in ADHD data. Therefore, these differences are further investigated and supported by statistical analysis and also validated using ML and DL techniques.

### Statistical analysis

The relationship between frontal and occipital lobes is observed, and the signals from the channels such as Fz, Fp1, Fp2, F3, F4, F7, F8, O1, and O2 are analyzed, and also “Alpha band” is analyzed because for the visual attention task, cognitive alertness better quantifies the ability of the children, and it is represented as a matrix. Using a heat map, the darker areas indicate a stronger influence and light area indicates a weaker influence.

### Coherence

The coherence matrix is measured in the processed data for the Alpha band, and Figure 7 is the coherence matrix of the two classes.

**Figure 8:**
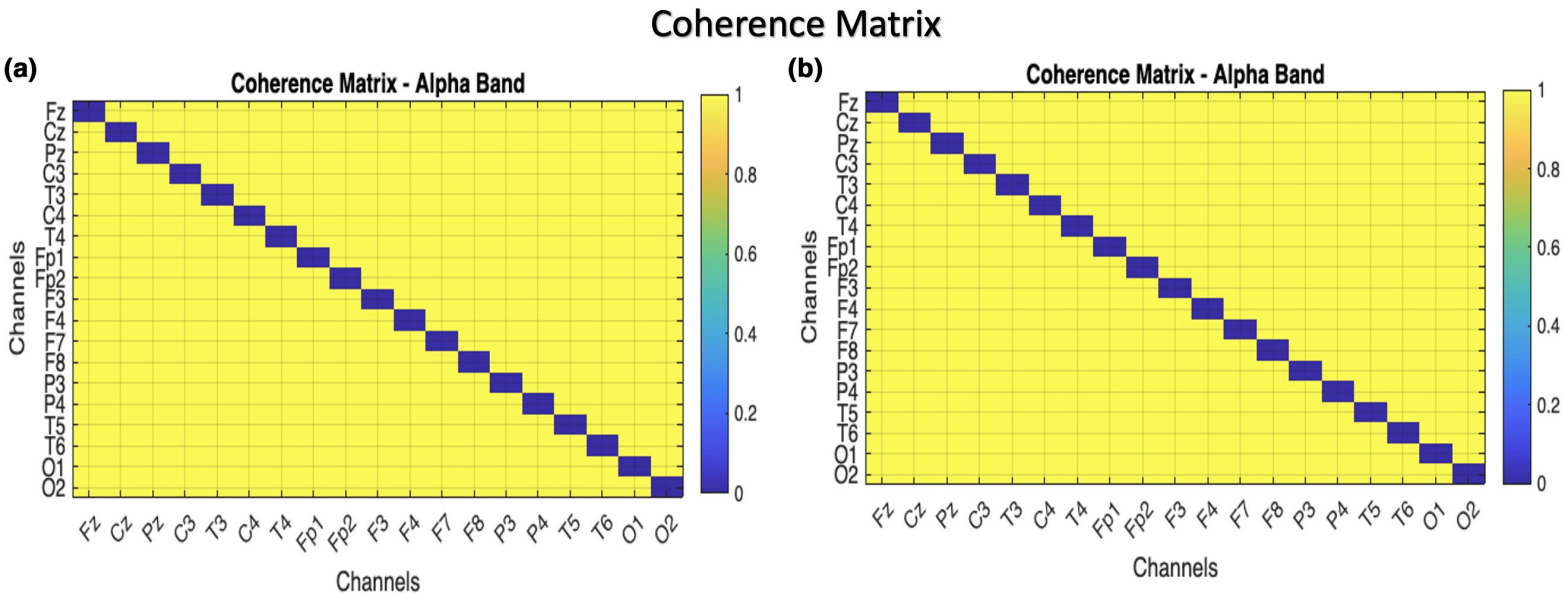
Comparison matrix of coherence between 19 channels of ADHD andc children to visualize the cohesive difference among channels. **(a)** ADHD children, **(b)** normal children.

The coherence matrices for children with ADHD and normal children, shown in Figure 8, are similar, but that should not be the case. This might occur because individual variability masks group-level differences.

### Granger causality

**Figure 9:**
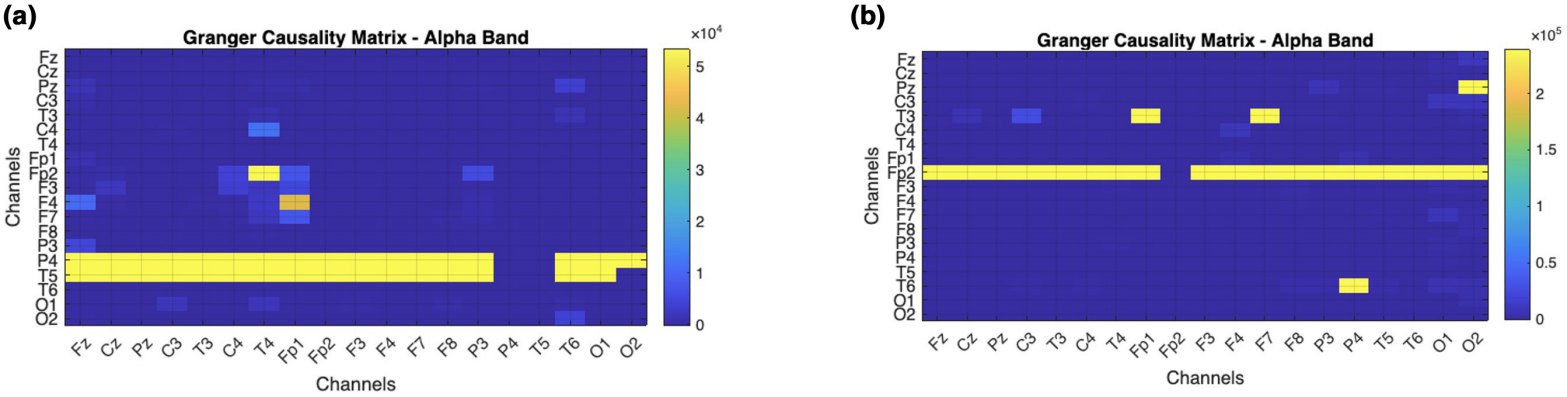
Comparison of granger causality matrix of 19 Channel to visualize the causal difference in channels and influence of occipital on frontal lobes. **(a)** ADHD children. **(b)** Normal children.

The Granger causality is measured on the processed data in the electrode channels, and the matrix is observed for both ADHD and normal children, as shown in Figure 9.

This technique indicates the causal influence of the occipital lobe on the frontal lobes, i.e., the influence of signals from channels O1 and O2 on the signals of channels such as Fz, Fp1, Fp2, F3, F4, F7, and F8. The regions are darker in the normal children causality matrix (b) compared to the ADHD children matrix (a), as in Figure 9. Applying granger causality, it is noticed that normal children have better connectivity patterns than ADHD children.

### PSI

**Figure 10:**
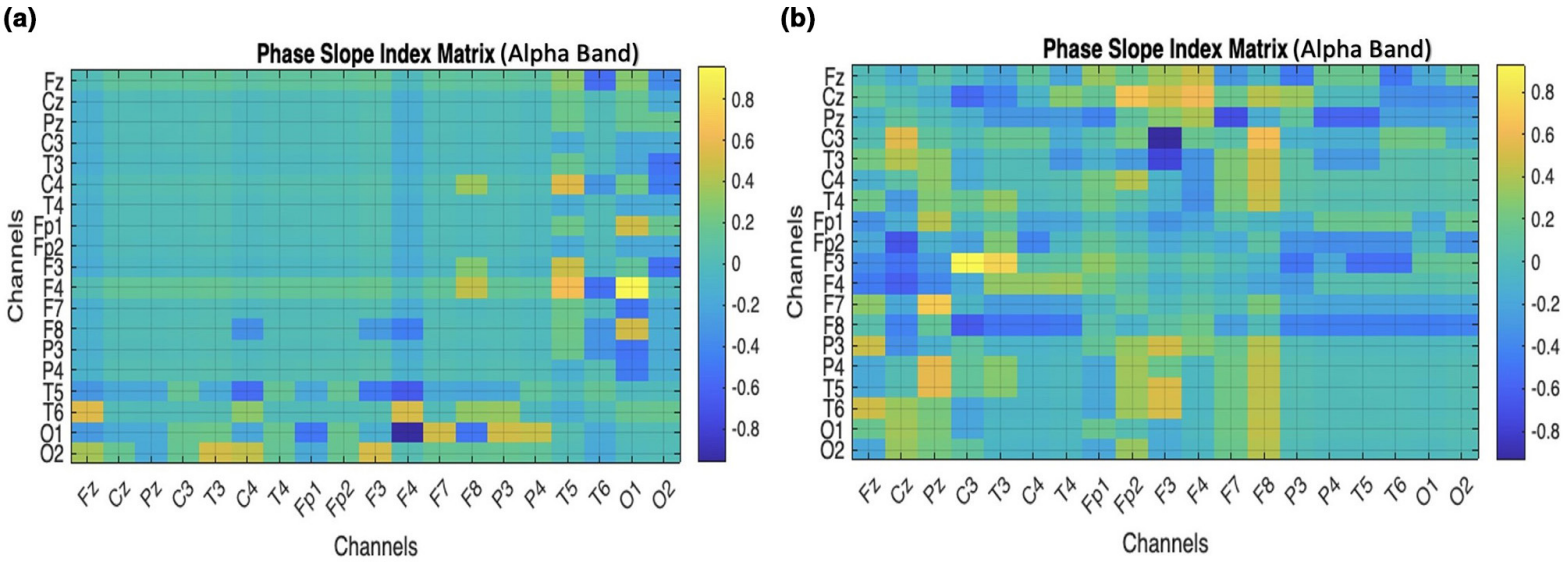
Comparison of Phase slope index to observe the influence of occipital on frontal through 19 channel electrodes. **(a)** ADHD children. **(b)** Normal children.

PSI is measured in processed data for the alpha band; the matrix is shown in Figure 10.

Using the PSI technique, the information flow direction over the frontal and occipital lobes is observed through phase relationship between the corresponding channels of the lobes are analyzed, and as in Figure 10 the matrix of children with ADHD (a) has lighter areas compared to channels in the matrix of normal children (b) as it has darker areas, which indicates more efficiency in strength of association between signals. Thus, it depicts that the phase synchronization is less in ADHD than in normal children.

### PDC

**Figure 11:**
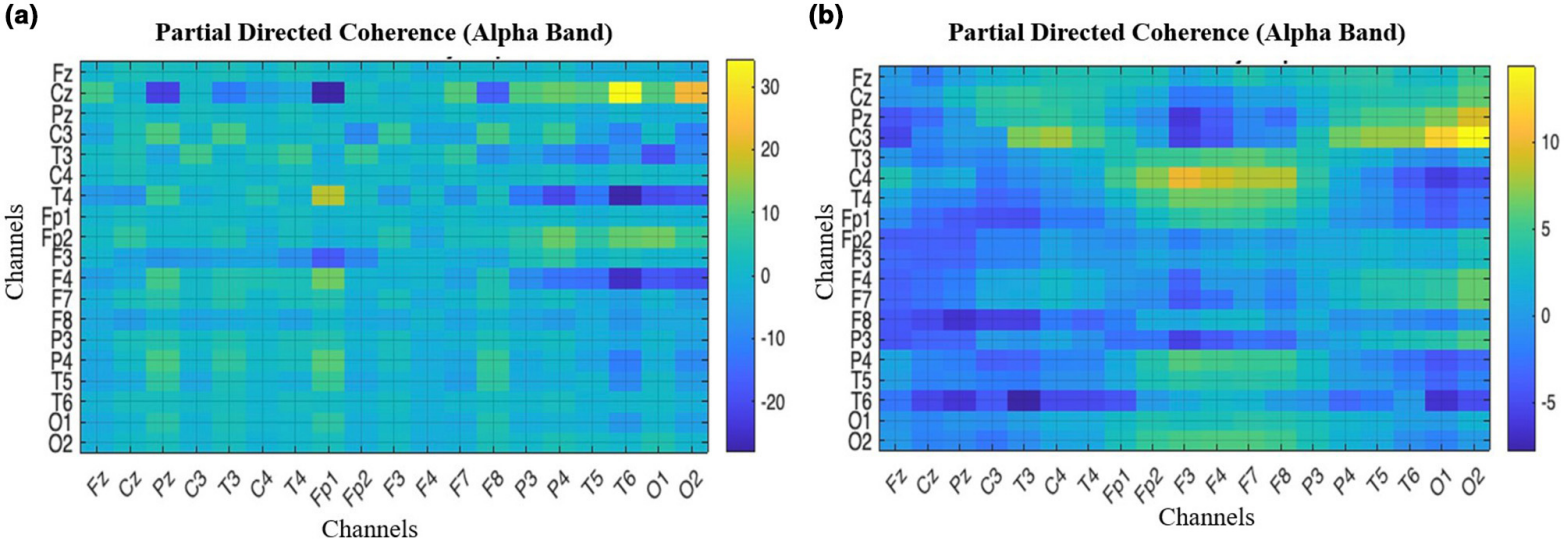
Partial directed coherence matrix is compared across 19 channel electrodes to understand the functional connectivity between frontal and occipital lobes. **(a)** ADHD children. **(b)** Normal children.

PDC measures the intensity of directed functional connectivity over the frontal and occipital lobes of the brain, and quantifies the influence and strength of the connection between them as shown in Figure 11. The influence of O1 and O2 on Fp1, Fp2, F3, and F4 is less in ADHD children compared to normal children.

### DTF

**Figure 12:**
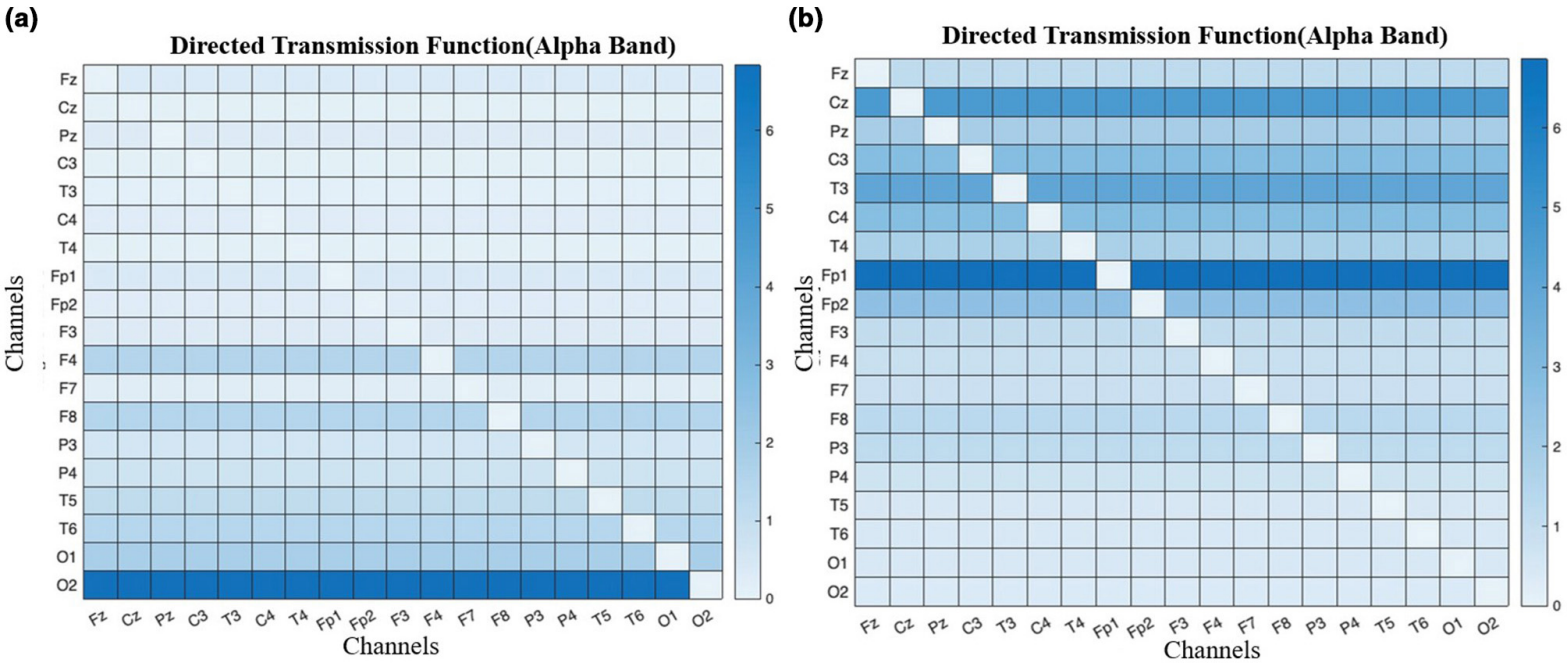
Representation of matrix comparison of directed transmission function between ADHD and Control by analyzing the relationship among channels directed transmission function through 19 channel electrodes. **(a)** ADHD children. **(b)** Normal children.

The directed transmission function in Figure 12 for children with ADHD and normal children indicates that the causal relationship is reduced in ADHD compared to normal children. The presence of darker regions between the frontal lobe and the occipital lobe channels, i.e., information flow transmission to Fp1 from O1 and O2 is higher for normal than for ADHD children, indicating that normal children have more efficient brain connectivity.

### Cognitive interpretation

By performing the visual attention task, the children were instructed to count the number of images given to them, lobes such as frontal and occipital are involved. From the EEG data collected the band power distributed over the frontal and occipital lobes is analyzed. As mentioned in Table 2, Alpha, Beta, and Delta are observed to be efficient in controls compared to children with ADHD as shown in Figures 7a, b. It was also investigated by analyzing the performance of the Alpha band and the influence of the occipital on the frontal through statistical analysis, as shown in Figures 8–12. However, existing studies also support the fact that the band power would be lower in ADHD than in control (Mart'nez-Briones et al., 2021; Kuc et al., 2017).

This study does not directly involve learning the therapeutic outcomes. However, since we analyze working memory patterns, we focus on specific bands such as Alpha, Beta, Delta, and Theta. We can hypothesize the impact of therapy for individuals with learning disability by concentrating on the band-specific patterns that agree with the four bands mentioned.

### Validation using ML and deep learning algorithms

ML and DL algorithms are validated using various metrics, and performance is analyzed for different algorithms. The stratified k-fold cross-validation has been used with k = 5, i.e., the dataset will be separated as 5 different subsets with the same proportion of data, and training will be performed for “k” iterations. So, for the value of k = 5, the iteration takes place 5 times, and each time one subset of the data is taken as test data with the rest as training data. For each iteration, the subsets of data for training and testing change.

#### SVM

**Figure 13:**
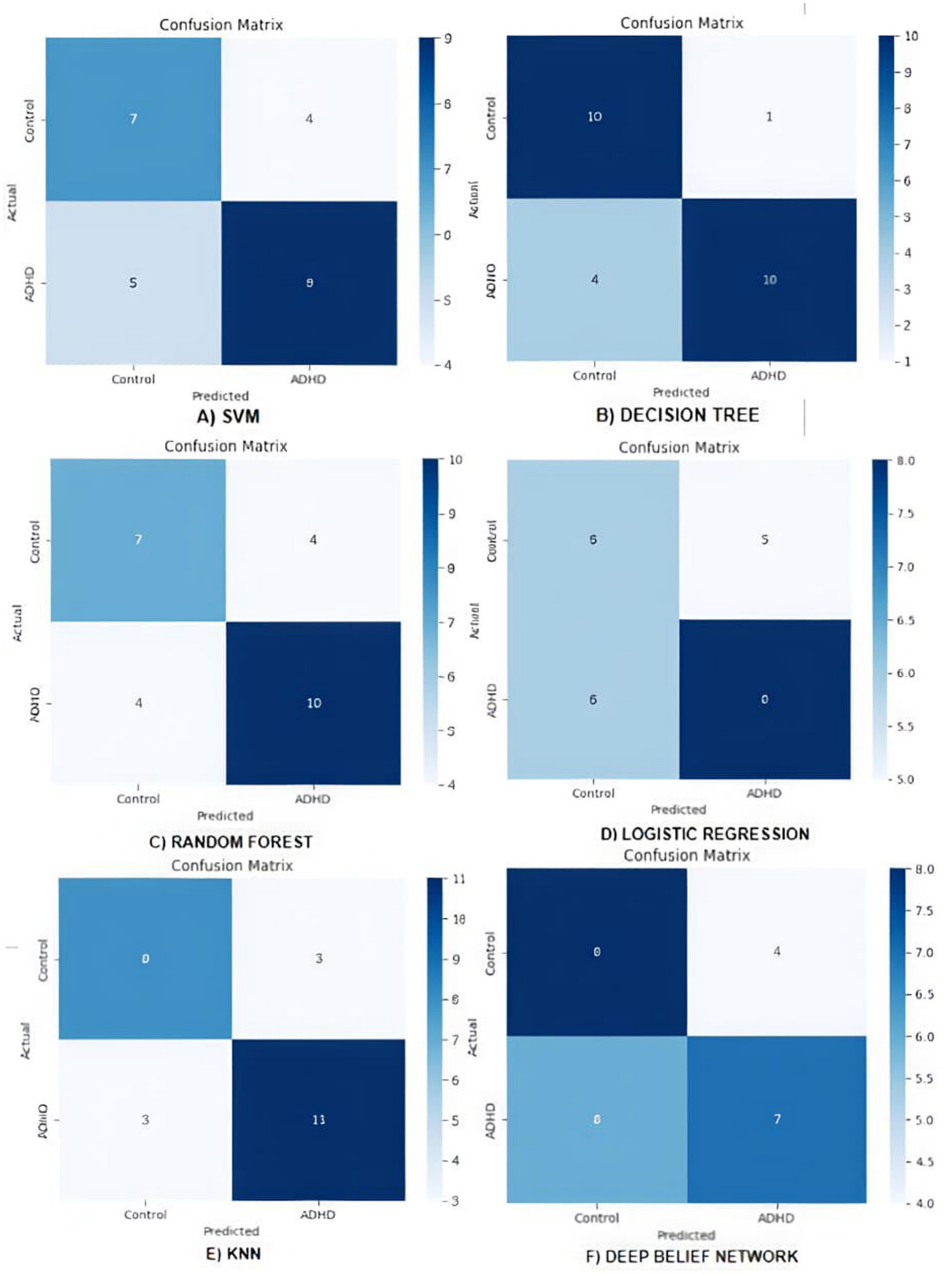
Confusion matrix for machine learning and deep learnign models **(A)** Support Vector Machine (SVM), **(B)** Decision Tree (DT), **(C)** Random Forest (RF), **(D)** Logistic Regression (LR), **(E)** KNN (K Nearest Neighbor, and **(F)** Deep Belief Network (DBN).

The algorithm is trained, and the confusion matrix is plotted as shown in Figures 13A, which is computed using the metrics in Table 3.

**Table 3:** Classification model performance comparison.

Model	Precision		Recall		F1 score		Accuracy
	ADHD	Controls	ADHD	Controls	ADHD	Controls	
SVM	58%	69%	64%	64%	61%	67%	64%
DT	67%	77%	73%	71%	70%	74%	63%
RF	50%	62%	55%	57%	52%	59%	66%
LR	50%	60%	45%	64%	48%	62%	67%
KNN	73%	79%	73%	79%	73%	79%	66%
DBN	82%	80%	75%	85%	78%	81%	89.7%

The confusion matrix classifies children with ADHD and normal using the SVM model with 0.73 of accuracy of the model, that is, 64% of cases in the cross-validation dataset are accurately identified. Control class with a precision score of 0.58 indicates that 58% of the children classified and categorized as normal are actually normal. The recall score of 0.69 indicates that the SVM model has correctly identified 69% of the normal class data and obtains a balanced precision–recall performance, indicated by an F1 score of 0.61. ADHD class with a precision score of 0.69 indicates that 69% of the cases classified by the model as children with ADHD are actually ADHD class. The recall score of 0.64 indicates that the 64% of the actual cases of children with ADHD are identified accurately, and the model achieves an F1 score of 0.67, reflecting a balance in handling both precision and recall.

#### DT

The algorithm is trained, and the confusion matrix is calculated as shown in Figure 13B. Using Table 3, the confusion matrix is computed. This algorithm classifies ADHD children and normal children with an accuracy of 63%. The ADHD class with precision score of 0.67 indicates that 67% of children are classified as ADHD are actually children with ADHD. The recall score of 0.73 indicates that the DT model has correctly identified 73% of the ADHD class data. The ADHD class obtains a balanced precision–recall performance, indicated by an F1 score of 0.70. Normal class with 0.77 precision score indicates that 77% of the cases classified by the model as normal children are actually normal. The recall score of 0.71 indicates that the 71% of the actual normal children cases are identified accurately, and the model achieves an F1 score of 0.74, reflecting a balance in handling both precision and recall.

#### RF

The algorithm is trained, and the confusion matrix is plotted as shown Figure 13C, which is computed using the metrics in Table 3.

The confusion matrix of the RF classifier model represents classification of children with ADHD and normal with 0.66 of model accuracy. Normal class with a precision score of 0.50 indicates that 50% of the children categorized as controls are actually normal. The recall score of 0.55 indicates that the model has accurately identified 55% of the normal class data and obtains a balanced precision–recall performance, indicated by an F1 score of 0.52. The class of children with ADHD with a precision score of 0.62 indicates that 62% of the cases were classified by the model as children with ADHD, but are actually children of ADHD class. The recall score of 0.57 indicates that the 57% of the actual children with ADHD cases are identified accurately, and the model achieves an F1 score of 0.59, reflecting a balance in handling both precision and recall.

#### LR

The algorithm is trained, and the confusion matrix is calculated as shown in Figure 13D, which is computed using the metrics in Table 3.

The confusion matrix that classifies the children with ADHD and normal using LR model with 0.55 of model accuracy, that is 55% of cases in the cross-validation dataset are accurately identified. Normal class of children with a precision score of 0.50 indicates that 50% of the children classified as controls are actually normal. The recall score of 0.55 indicates that the random forest classifier model has correctly identified 55% of the control class data and obtains a balanced precision–recall performance, indicated by an F1 score of 0.48. ADHD class with precision score of 0.60 indicates that 60% of the children cases were classified by the model as children with ADHD but are actually ADHD class children. The recall score of 0.64 indicates that the 64% of the actual children of ADHD cases are identified accurately, and the model achieves an F1 score of 0.62, reflecting a balance in handling both precision and recall.

#### KNN

The algorithm is trained, and confusion matrix is calculated as shown in Figure 13E, which is computed using the metrics in Table 3. The confusion matrix of the KNN represents the classification of children with ADHD and normal with 0.66 of model accuracy. Normal class with a precision score of 0.73 indicates that 73% of the children categorized as controls are actually normal. The recall score of 0.73 indicates that the model has accurately identified 73% of the control class data and obtains a balanced precision–recall performance, with an F1 score of 0.73. ADHD class with precision score 0.79 indicates that 79% of the children classified by the model as children with ADHD are actually ADHD. The recall score of 0.79 indicates that the 79% of the actual children of ADHD cases are identified accurately, and the model achieves an F1 score of 0.79, which reflects a balance in handling both precision and recall.

#### DBN

This deep learning algorithm is trained, and the confusion matrix is calculated as shown in Figure 13F. Using the metrics in Table 3, the confusion matrix is computed. DBN classifies the children with ADHD and control children with a model accuracy of 89.7%. For the Normal class, a precision score of 0.82 indicates that 82% of children are classified as control are actually normal. The recall score of 0.75 shows that the DBN model correctly identified 75% of the normal class data resulting in a balanced precision–recall performance with an F1 score of 0.78.

For the ADHD class, a precision score of 0.79 indicates that 79% of the cases classified by the model as ADHD are correctly identified. The recall score of 0.85 demonstrates that 85% of the actual ADHD cases are identified accurately, and the model achieves an F1 score of 0.81, reflecting a balance in handling both precision and recall.

### Model behavior comparison

This section presents a comparison of model performances across different sequence-based architectures applied to classification. The evaluation was carried out by comparing the metrics of the six algorithms—SVM, RF, LR, KNN, DT, and DBN—as mentioned in Table 3. Among the experimented models, DBN emerged as the effective model, achieving a fair accuracy of 89.7% for the driven IEEE dataset. It demonstrated a strong balance across all metrics. For example, DBN achieved f1 scores of 0.78 for class 0 and 0.81 for class 1, indicating the reliability of the model in correctly identifying in both classes.

**Figure 14:**
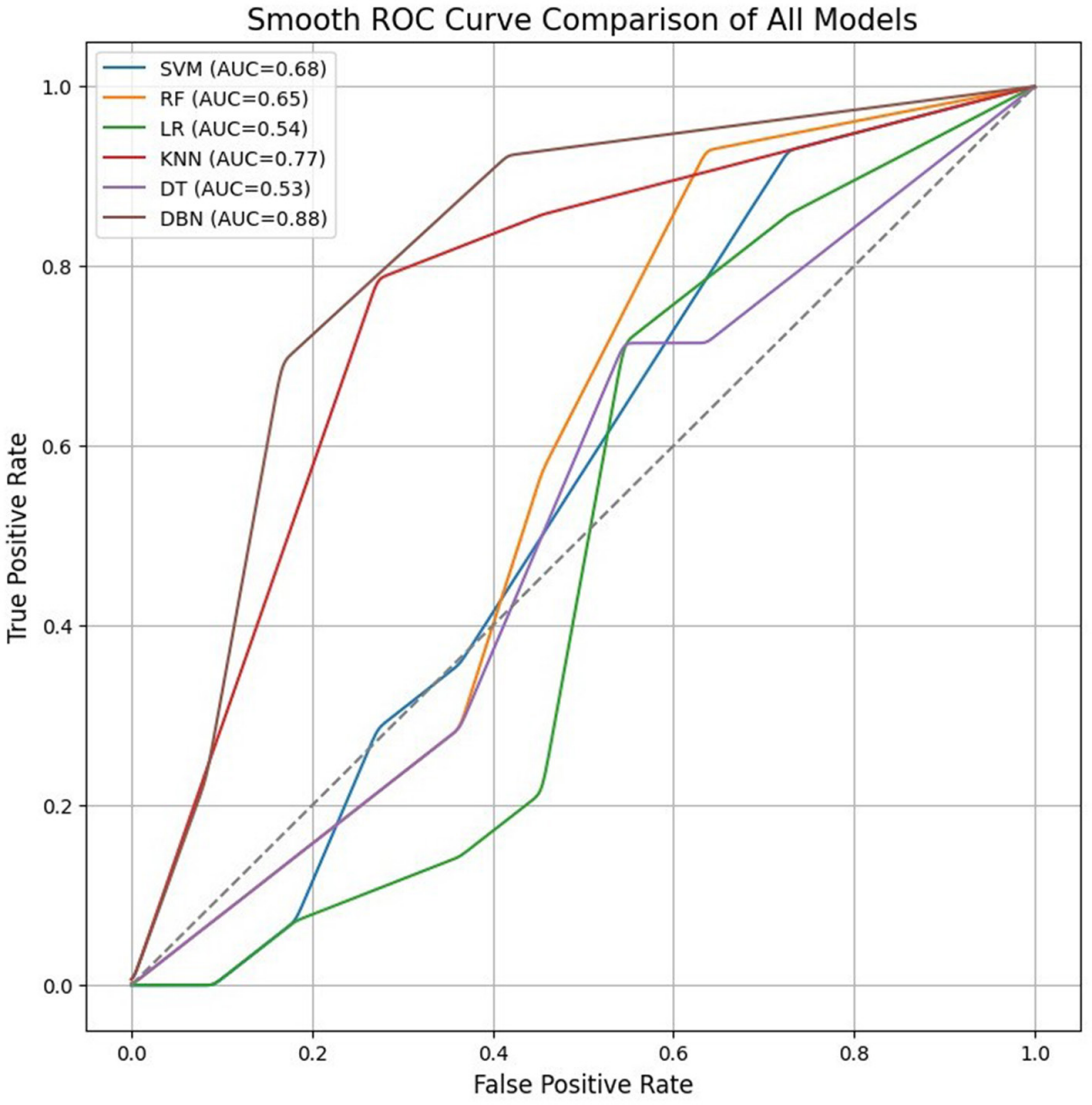
AUCROC curve for machine learning and deep learning models.

Table 4 enlists the performance comparison between existing research studies against our findings. It has been observed that our approach provides 64% accuracy for SVM, while a similar study using EEG data has detected dyslexia with an accuracy of 62.4%. Other similar studies involved the detection of ADHD with a different methodology using feature fusion. For the given methodology implemented in this research, although the conventional ML algorithms fared well in terms of exhibiting a reasonable classification accuracy, it has been observed that the DBN provided an increase and outperformed accuracy against the trend set and baseline ML models with the given current data availability. As the data increase, there is a fair chance of expecting the models to learn well thereby, contributing to increased classification accuracy. The studies employing DBN have been used for fMRI and MRI modalities to date. However, we have attempted to make use of DBN for EEG data study with an intention to perform investigations on working memory patterns. Although, LR is obtained as an optimal model in comparison with other experimented ML models, applying a deep-learning model like DBN helped to witness an outperformed overall classification accuracy measuring 90% against both trend set and baseline models. AUC-ROC curve distinguishes the significant comparison of all the experimented models, and DBN achieving maximum area indicating its best performance than other models, as shown in Figure 14.

**Table 4:** Comparison of research finding with existing study.

Model	Existing research accuracy (in percent)	Findings accuracy (in percent)
SVM	62.4	64
DT	73.57	63
RF	83.2	66
KNN	82.95	66
DBN	–	89.7

## Conclusion

Investigation of working memory patterns using quantitative techniques has effectively supported the differentiation of children with learning disabilities from normal children. EEG signals of children driven by IEEE Dataport were acquired by assigning visual attention tasks to test their ability toward cognitive processes such as attention and thinking. Employing these data in conjunction with ML algorithms has contributed well to investigating the working memory patterns of learning disabilities. Using quantitative brain connectivity measures, the strength of connection between the frontal and occipital lobes is analyzed, as they are responsible for processing the visual attention task of counting images. The outcome of the study indicates that brain connectivity in normal children is better than in children with learning disability with evidence of quantitative analysis of brain connectivity parameters. This finding is also validated using algorithms such as *SVM with 64%, RF with 66%, LR with 67%, KNN with 66%, DT with 63%*, and *DBN with 89.7%* model accuracy. From which it is observed, LR is better than other experimented ML models but still with less accuracy intended to employ deep learning model DBN for the stated approach.

In this research, EEG signals are analyzed using statistical methods specifically for the Alpha frequency band of the frontal and occipital lobes rather than using VMD and HT for mode conversion to extract features and a further classification of ADHD and normal children (Khare and Acharya, 2023). The metric shown in Tables 3, 4 clarifies that among ML models, LR performs fairly and is outperformed by the DBN deep learning model.

This concept of ideology can also be applied to the real-time dataset obtained by assigning a task to access cognitive skills because the analysis of cognitive skills helps measure learning disability. But for a real-time dataset, the processes of pre-processing might differ as the data would be obtained after undergoing additional techniques to remove artifacts to clean and prepare the data.

This study contributed to the advancement of therapeutic intervention by identifying minor changes in remedial children as they are outliers and their minor improvements are not voiced well. Through the analysis of brain connectivity parameters between children with learning disabilities and normal children, the status of the remedial child is mapped. Furthermore, hybrid models can also be used to improve the efficiency of machine learning models in classifying classes to gain better accuracy.

## Data Availability

Publicly available datasets were analyzed in this study. This data can be found here: The authors clarify that ethically the datasets used in this research are not live data acquired in real-time from the children, instead they are driven from IEEE Dataport, an open-access dataset repository publicly available for research investigation. https://ieee-dataport.org/open-access/eeg-data-adhd-control-children.
